# The mediating role of loneliness in the effect of social media addiction on aesthetic procedures in women

**DOI:** 10.1590/1806-9282.20241129

**Published:** 2025-05-02

**Authors:** Eda Yakit AK, Mehmet Ali Şen, Özden Tandoğan

**Affiliations:** 1 Dicle University, Atatürk Health Services Vocational School – Diyarbakır, Turkey.; 2 Istanbul Arel University, Faculty of Health Sciences, Department of Nursing – İstanbul, Turkey.

**Keywords:** Social media, Social media addiction, Esthetics, Loneliness

## Abstract

**OBJECTIVE:**

The aim of this study was to determine the mediating role of loneliness in the effect of women's social media addiction on aesthetic procedure behavior.

**METHODS:**

The study was carried out with a total of 1,166 women. The data were evaluated by correlation and SPSS PROCESS macro 4 regression analysis with the Introductory Information Form, Social Media Addiction Scale-Adult Form, Social Media and Changing Perception of Aesthetic Procedures in Society Scale, and Ruls-6 Loneliness Scale.

**RESULTS:**

When the mediating role of loneliness was examined in the effect of social media addiction on having aesthetic procedures, it was determined that both social media addiction (**β**=0.481) and loneliness (**β**=0.075) significantly positively affected the perception of having social media aesthetic procedures. A positive relationship was determined between Social Media Addiction Scale-Adult Form, Social Media and Changing Perception of Aesthetic Procedures in Society Scale total score, sub-factor mean scores, and Ruls-6 Loneliness total scores (p<0.001).

**CONCLUSION:**

It was found that loneliness had a low-level effect on the effect of social media addiction on aesthetic behavior.

## INTRODUCTION

Societies all over the world have been affected by the changes in social media, and the positive and negative effects of social media have been discussed in this article. Among the positive effects of social media on individuals is the ability of the masses to come together in a virtual environment, reducing the feeling of loneliness, providing an environment for fast access to information, and sharing ideas^1^. On the contrary, many negative emotions are caused by the misuse of social media. Perfect lives shared on social media, which are often unrealistic, cause individuals to question their lives and feel a sense of inadequacy^2^. One of these deviations from reality is beauty. The perfect face and body lines shared and appreciated on social media have started to increase the importance people attach to their bodies or all their physical characteristics^3^.

The increasing influence of social media on daily life increases the tendency of users to compare and evaluate themselves with other people^4^. Social media interactions shape people's communication, interests, and moods, and this plays a decisive role in their aesthetic perceptions. Images of perfect lives that are frequently shared on social media platforms can cause individuals to question their own lives and experience a sense of inadequacy^3^. This situation paves the way for the concepts of aesthetics, beauty, and the perfect body to be easily communicated through social media and direct individuals to aesthetic procedures^3^. Studies examining the relationship between social media use and mental health problems have revealed that it may be associated with problems such as anxiety and depression. In addition, research also shows that social media use differs according to gender and that women are more affected than men^5,6^. Women's intense addiction to social media and the filtered and flawless beauty image they are constantly exposed to may cause them to seek perfection in their own bodies. It is thought that especially women may associate coping with the feeling of loneliness, social acceptance, socialization, and appreciation with their appearance^6^. For this reason, it is more possible to have aesthetic procedures that are not needed. In recent years, social media has become an important tool that shapes individuals’ lives and body perceptions^3,5^. Especially women tend to feel inadequate due to the perfect beauty standards they frequently encounter on social media platforms. This may have increased the tendency toward aesthetic procedures. The perceptions of ideal beauty that women are exposed to through social media may trigger bodily dissatisfaction and the search for aesthetic intervention. Social media causes individuals to compare themselves with others and feel low self-esteem as a result of these comparisons. In this regard, this study aims to examine the mediating role of loneliness in the effect of social media addiction on the perception of having aesthetic procedures in women. It is thought that the findings will contribute to understanding the complex relationships between social media use, body perception, and mental health.

## METHODS

This descriptive and cross-sectional study was conducted with an online survey in May–June 2024. Before starting data collection, necessary permissions were obtained from the Non-Interventional Clinical Research Ethics Committee (ethics number: E14679147-663.05-710841). The population of the study consisted of women over the age of 18 years who use at least one social media application. Since maximum diversity was targeted in the study, it was completed with 1,166 women. The principles of the Declaration of Helsinki were complied with in the study. Research data were collected through an online form (Google Form). Participants were invited to participate in the study through the researchers’ social media channels (LinkedIn, Instagram, Facebook, and WhatsApp). An informed consent form on the first page informed women about the research's purpose and confidentiality. Those who selected "I agree" could proceed, while those who chose "I do not agree" were unable to participate.

Social Media Addiction Scale-Adult Form (SMAS-AF): The scale is a 5-point Likert-type scale consisting of 20 items and two sub-dimensions (virtual tolerance and virtual communication). The highest score that can be obtained from this scale is 100, and the lowest score is 20. The Cronbach's alpha reliability coefficient of the scale was found to be 0.94^7^.

Social Media and Changing Perception of Aesthetic Procedures in Society Scale (SMCPAPSS): The scale consists of 16 items and 4 sub-dimensions. There are no reverse items in the scale. The scoring of this scale varies between 18 and 90. The Cronbach's alpha reliability coefficient of the scale was found to be 0.90^8^.

Ruls-6 Loneliness Scale: The scale is a 4-point Likert-type scale consisting of six items and one sub-dimension. The lowest score is 6, and the highest score is 24. The Cronbach's alpha reliability coefficient of the scale was found to be 0.84^9,10^.

In our study, the Cronbach's alpha value of the SMAS-AF was 0.93, the SMCPAPSS was 0.95, and the Ruls-6 Loneliness Scale was 0.85.

In descriptive statistics, mean, standard deviation, minimum, and maximum values were given for numerical variables, while number and percentage values were given for categorical variables. A t-test was performed to test whether there was a significant difference between the groups. Differences between three or more groups were analyzed by one-way analysis of variance (ANOVA). SPSS PROCESS macro 4 regression analysis was used.

## RESULTS

The mean age of the women who participated in the study was 29.17±10.01 years (min–max: 18–65 years). Among the women, 49.7% were between the ages of 18 and 25, 41.2% were university graduates or higher, 66.3% were single, 70.8% were employed, and 49.1% had a poor income. Of the women, 40.4% reported that they spent 5 h or more on social media per day (Table 1).

**Table 1:** Socio-demographic characteristics of women and their relationship with scales.

Variables (n: 1,166)		n	%	SMAS-AF	Test and significance	SMCPAPSS	Test and significance	Ruls-6 Loneliness	Test and significance
				$\bar{X}$ ±*SD*		$\bar{X}$ ±*SD*		$\bar{X}$ ±*SD*	
Age									
	18–25 (1)	580	49.7	54.51±16.10	F: 10.830 **p: 0.000** **(1-2, 1-3, 1-4)**	38.74±15.28		14.54±4.23	F: 8.935 **p: 0.000** **(1-3, 1-4, 2-4)**
	26–35 (2)	303	26.0	51.24±13.59		38.36±15.41	F: 0.978	14.31±3.80	
	36–45 (3)	191	16.4	50.77±14.90		38.30±14.21	p: 0.402	13.36±4.07	
	≥46 (4)	92	7.9	46.37±14.44		35.83±15.10		12.54±4.31	
Education									
	≤Secondary school graduate (1)	220	18.9	49.00±16.73	F: 5.907	38.25±15.11	F: 0.118	13.50±4.51	F: 3.226
	High school graduate (2)	466	40.0	54.00±16.34	**p: 0.003**	38.12±15.26	p: 0.889	14.35±4.01	**p: 0.040**
	≥University graduate (3)	480	41.2	51.93±14.83	**(2-1)**	38.60±15.03		14.20±4.08	**(2-1)**
Marital status									
	Married	393	33.7	49.57±15.50	t: −4.741	37.87±15.01	t: y0.759	13.14±4.05	t: −5.866
	Single	773	66.3	53.85±15.89	**p: 0.000**	38.58±15.19	p: 0.448	14.63±0.10	**p: 0.000**
Employment status									
	Not working	340	29.2	52.02±15.46	t: y0.570	39.28±15.18	t: 1.354	13.70±3.90	t: −2.288
	Working	826	70.8	52.569±16.01	p: 0.569	3.96±15.10	p: 0.176	14.30±4.23	**p: 0.022**
Income status									
	Poor (1)	573	49.1	52.90±16.33	F: 0.733	38.92±15.85	F: 1.254	14.67±4.30	F: 10.003
	Medium (2)	454	38.9	51.79±15.45	p: 0.481	37.47±14.48	p: 0.286	13.69±3.94	**p: 0.000**
	Good (3)	139	11.9	52.33±15.13		38.83±14.09		13.35±3.8	**(1-2, 1-3)**
Time spent on social media Daily									
	0–2 h (1)	297	25.5	46.27±15.03	F: 50.380	34.32±13.59	F: 17.334	13.22±4.28	F: 16.919
	4–6 h (2)	398	34.1	51.87±14.79	**p: 0.000**	38.40±14.96	**p: 0.000**	13.88±3.80	**p: 0.000**
	≥5 h (3)	471	40.4	56.72±16.16	**(2-1, 3-1, 3-2)**	40.83±15.67	**(2-1, 3-1, 3-2)**	14.92±4.21	**(3-1, 3-2)**

F: Anova test, t: Student's t-test, 1-2-3-4: groups with differences. SD: standard deviation. SMAS-AF: Social media addiction scale, SMCPAPSS: Social Media and Changing Perception of Aesthetic Procedures in Society Scale, Ruls-6 Loneliness: Ruls-6 Loneliness Scale. Statistically significant values are indicated in bold.

**Figure 1:**
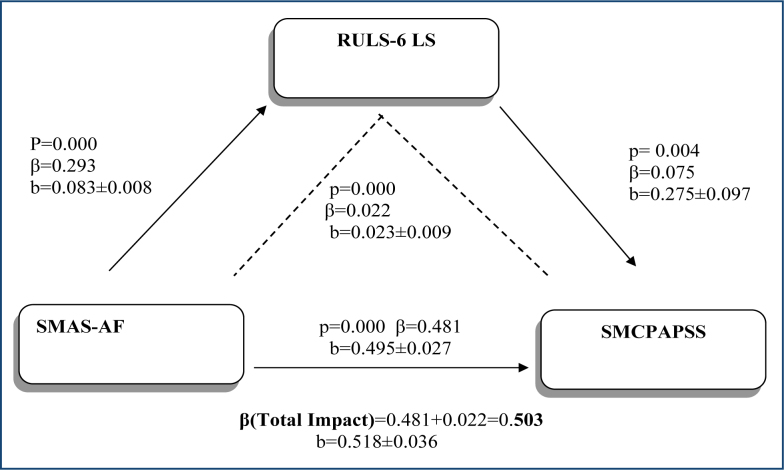
Research model. SMAS-AF: Social Media Addiction Scale; SMCPAPSS: Social Media and Changing Perception of Aesthetic Procedures in Society Scale; Ruls-6 Loneliness: Ruls-6 Loneliness Scale.

The effect of the mediating role of loneliness on the effect of women's social media addiction status on the perception of having social media aesthetic procedures is shown in Figure 1. According to the results of the analysis, both social media addiction (β=0.481) and loneliness (β=0.075) have a significant positive effect on the perception of having social media aesthetic procedures. According to another finding, loneliness has a mediating role in the effect of social media addiction on the perception of having social media aesthetic procedures (β=0.022), and this role has a low level of mediation effect. Loneliness further increases the positive effect of social media addiction status on having social media aesthetic procedures (β=0.503).

It was determined that the participants in our study were moderately dependent on the average score they received from the social media addiction scale (52.41±14.69), the average score they received from the social media aesthetic perception scale, and the effect of social media on the desire to have aesthetic procedures at a low level (38.35±15.13) and the score they received from the Ruls-6 Loneliness Scale and they felt loneliness at a moderate level (14.13±4.15).

It was found that there was a relationship between the total scores and sub-factors of SMAS-AF and SMCPAPSS and the total scores of the Ruls-6 Loneliness Scale, and the relationship levels are given in Table 2 (p<0.05).

**Table 2:** The relationship between Social Media Addiction Scale-Adult Form, Social Media and Changing Perception of Aesthetic Procedures in Society Scale total score and sub-factors mean scores, and Ruls-6 Loneliness total scores.

		SMAS-AF total score	SMAS-AF Sub 1	SMAS-AF Sub 2	SMCPAPSS total score	SMCPAPSS Sub 1	SMCPAPSS Sub 2	SMCPAPSS Sub 3	SMCPAPSS Sub 4	Ruls-6 LS total score
SMAS-AF total score	**r**	1								
	p									
SMAS-AF sub-factor 1	r	**0.926** **	1							
	p	0.000								
SMAS-AF sub-factor 2	r	**0.928** **	**0.718** **	1						
	p	0.000	0.000							
SMAS Total score	r	**0.503** **	**0.478** **	**0.453** **	1					
	p	0.000	0.000	0.000						
SMCPAPSS sub-factor 1	r	**0.486** **	**0.470** **	**0.430** **	**0.952** **	1				
	p	0.000	0.000	0.000	0.000					
SMCPAPSS sub-factor 2	r	**0.402** **	**0.357** **	**0.387** **	**0.842** **	**0.735** **	1			
	p	0.000	0.000	0.000	0.000	0.000				
SMCPAPSS sub-factor 3	r	**0.384** **	**0.390** **	**0.322** **	**0.786** **	**0.661** **	**0.580** **	1		
	p	0.000	0.000	0.000	0.000	0.000	0.000			
SMCPAPSS sub-factor 4	r	**0.445** **	**0.412** **	**0.413** **	**0.862** **	**0.737** **	**0.656** **	**0.675** **	1	
	p	0.000	0.000	0.000	0.000	0.000	0.000	0.000		
Ruls-6 LS	r	**0.294** **	**0.306** **	**0.238** **	**0.216** **	**0.223** **	**0.137** **	**0.180** **	**0.184** **	1
Total score	p	0.000	0.000	0.000	0.000	0.000	0.000	0.000	0.000	

r=Pearson correlation coefficient,

** p<0.001, SMAS-AF: Social media addiction scale, SMCPAPSS: Social Media and Changing Perception of Aesthetic Procedures in Society Scale, Rulls-6 Loneliness: Rulls-6 Loneliness Scale.

r= correlation values. Significant values are those in bold and dark-colored bold. SMA S-AF Sub 1: Social media addiction scale subfactor virtual tolerance. SMA S-AF Sub 2: Social media addiction scale subfactor virtual communication. SMCPA PSS Sub 1: Social Media and Changing Perception of Aesthetic Procedures in Society Scale subfactor need. SMCPA PSS Sub 2: Social Media and Changing Perception of Aesthetic Procedures in Society Scale subfactor domain. SMCPA PSS Sub 3: Social Media and Changing Perception of Aesthetic Procedures in Society Scale subfactor accessibility. SMCPA PSS Sub 4: Social Media and Changing Perception of Aesthetic Procedures in Society Scale subfactor visibility.

## DISCUSSION

We found that women with higher levels of education had lower social media addiction but similar levels of loneliness. Burkovik et al. explained the effect of education level on social media use by the development of individuals’ cognitive skills and self-control mechanisms. It has been reported that highly educated women are more resistant to the negative effects of social media and have a lower risk of developing addiction^11^. In our study, it was also observed that advanced age decreased social media addiction and desire for aesthetic procedures in women. This result may indicate that women are more concerned with menopause-related problems^12^.

On the other hand, the similar loneliness levels of women, regardless of education level, suggest that loneliness may be related to more complex social and psychological factors independent of social media use. In conclusion, the current research findings point to the protective role of education level on social media addiction, while its effect on loneliness is more limited.

In this study, a significant positive correlation was found between women's social media addiction (SMAS-AF) and their perception of social media aesthetic procedures (SMCPAPSS). Accordingly, as women's social media addiction levels increased, their perception of having aesthetic procedures through social media also increased. Boursier et al. examined the effect of social media use on body dissatisfaction and plastic surgery tendency in women. In the study, it was determined that women who received insufficient attention and negative feedback on social media were concerned about their appearance and turned to aesthetic interventions^13^. This supports the relationship between social media addiction and the perception of having aesthetic procedures. Similarly, McComb and Mills emphasized that perfect beauty images on social media can negatively affect individuals’ perceptions of their own bodies, which may increase the desire to have aesthetic procedures^3^. It can also be used to increase the sexual attractiveness of women with their bodies and to ensure that they are recognized^14^. In this context, with the increasing use of social media, it is observed that women focus more on their physical appearance and increase their plastic surgery preferences in order to ensure social acceptance.

The decrease in daily social communication with the increase in social media use causes individuals to feel lonely^15^. It is quite remarkable that women turn to aesthetic procedures to cope with loneliness. According to the results of the research, loneliness was found to have a low-level mediating role in the effect of social media addiction on having aesthetic procedures. In a study, it was determined that women who received insufficient attention and negative feedback on social media tended to undergo aesthetic interventions. It was also found that loneliness played a low level mediating role in this relationship^13^. In the results of this research, we found that social media addiction and loneliness have significant effects on women's perception of having aesthetic procedures. Understanding the complex relationships between social media use, body perception, loneliness, and psychological factors such as having aesthetic procedures is critical for women's mental health and well-being. Due to the growing influence of social media, women are more prone to constantly compare and evaluate themselves, resulting in increased feelings of body dissatisfaction and loneliness.

### Limitations

As this was a cross-sectional study, it may not have been possible to identify causal relationships between variables fully. A longitudinal research design would have provided a better understanding of how variables change over time and the dynamics in the relationships. The sample consists of women only. A comparative study including men would have provided an opportunity to examine the relationships between social media use, aesthetic procedures, and loneliness in a more in-depth gender context. The data collection process was carried out only through an online survey. The fact that the study was conducted on a Turkish sample only limits the generalizability of the results to different cultural contexts. Due to these constraints, there are some limitations regarding the generalizability of the findings and the full elucidation of the causal relationships between the variables.

## CONCLUSION

It is important to examine the relationship between social media use, body perception, and mental health more comprehensively and holistically. Specifically, we need to gain a better understanding of the psychological needs and challenges faced by women in this context. Additionally, it is important to investigate the psychological well-being of women who seek aesthetic procedures and the motivations behind their choices. Understanding the reasons why some women pursue unnecessary or excessive aesthetic procedures can help us avoid situations that may lead to regret.

## References

[1] Ostic D, Qalati SA, Barbosa B, Shah SMM, Galvan Vela E, Herzallah AM (2021). Effects of social media use on psychological well-being: a mediated model. Front Psychol.

[2] Tiggemann M, Hayden S, Brown Z, Veldhuis J (2018). The effect of Instagram "likes" on women's social comparison and body dissatisfaction. Body Image.

[3] McComb SE, Mills JS (2021). Young women's body image following upwards comparison to Instagram models: the role of physical appearance perfectionism and cognitive emotion regulation. Body Image.

[4] Kıran S, Küçükbostancı H, Emre İE (2020). Sosyal medya kullanımının kişiler üzerindeki etkilerinin incelenmesi. Bilişim Teknolojileri Dergisi.

[5] Karim F, Oyewande AA, Abdalla LF, Chaudhry Ehsanullah R, Khan S (2020). Social media use and its connection to mental health: a systematic review. Cureus.

[6] Uslu M (2021). Türkiye'de sosyal medya bağımlılığı ve kullanımı araştırması. Turk Acad Res Rev.

[7] Şahin C, Yağcı M (2017). Sosyal medya bağımlılığı ölçeği-yetişkin formu: geçerlilik ve güvenirlik çalışması. KEFAD.

[8] Türk GD, Bayrakçı S (2019). Sosyal medya ve toplumda değişen estetik işlem yaptırma algısı. Bilişim Teknolojileri Online Dergisi.

[9] Wongpakaran N, Wongpakaran T, Pinyopornpanish M, Simcharoen S, Suradom C, Varnado P (2020). Development and validation of a 6-item Revised UCLA Loneliness Scale (RULS-6) using Rasch analysis. Br J Health Psychol.

[10] Inanç A, Eksi H (2022). Adaptation of RULS-6 loneliness scale (6-item short form) into Turkish: a validity and reliability study. Contemp Educ Res J.

[11] Burkovik T, Bjelobrk D, Turk T (2021). The relationship between social media addiction, loneliness, and educational level among women. Druš Istraž.

[12] Zangirolami-Raimundo J, Sorpreso ICE, Rebouças CMP, Bezerra PCL, Costa LMPRD, Baracat EC (2023). Depression in women in climacteric period: a brief review. Rev Assoc Med Bras (1992).

[13] Boursier V, Gioia F (2020). Do selfie-expectancies and social appearance anxiety predict adolescents’ problematic social media use?. Comput Hum Behav.

[14] Firmino Murgel AC, Santos Simões R, Maciel GAR, Soares JM, Baracat EC (2019). Sexual dysfunction in women with polycystic ovary syndrome: systematic review and meta-analysis. J Sex Med.

[15] Cataldo I, Lepri B, Neoh MJY, Esposito G (2021). Social media usage and development of psychiatric disorders in childhood and adolescence: a review. Front Psychiatry.

